# Precision medicine for older adults with multimorbidity through a whole-person and palliative care approach

**DOI:** 10.3389/fmed.2026.1892671

**Published:** 2026-09-28

**Authors:** Oladunni Oyedotun, Oluwafunmibi Anjorin, David Dosa, Alok Kapoor, Jerry Gurwitz, Ann Berger, Hawa O. Abu

**Affiliations:** 1 Division of Geriatric Medicine, University of Massachusetts Chan Medical School, Worcester, MA, United States; 2 Division of Hospital Medicine, University of Massachusetts Chan Medical School, Worcester, MA, United States; 3 Division of Gerontology, Geriatrics and Palliative medicine, University of Maryland at Baltimore School of Medicine, Baltimore, MD, United States

**Keywords:** multimorbidity, older adults, precision medicine, spirituality, whole person care

## Abstract

As populations age globally, multimorbidity, defined as the coexistence of two or more chronic conditions, has become highly prevalent. While precision medicine holds promise for tailoring interventions based on genetics, environment, lifestyle, and other individual characteristics, its application in older adults with multimorbidity remains limited largely narrowly biomedical, disease-specific, and insufficiently aligned with palliative care principles. Such approaches are poorly suited to the complexity, vulnerability, prognostic uncertainty, and existential challenges characteristic of aging with multimorbidity, particularly in the context of serious illness. We argue that precision medicine must evolve into precision whole-person care by explicitly integrating palliative care principles, including goal-concordant care, relief of suffering, and preservation of dignity. This expanded model incorporates biological, psychosocial, and spiritual domains as measurable and actionable determinants of health. To promote a truly patient-centered, whole-person approach to precision medicine, all the dimensions of wellbeing should be recognized and integrated as key components of precision care. We synthesize evidence on multimorbidity, precision palliative care, and aging, and propose a multidimensional biopsychosocial–spiritual framework to guide the implementation of precision medicine in older adults, examine ethical and clinical implications; and suggest future research directions. Precision medicine from a whole person approach that recognizes and incorporates psychosocial-spiritual dimensions of wellbeing may better align care with patient values, enhance dignity, reduce burdensome or non-beneficial interventions, and optimize outcomes that matter most to older adults living with multiple chronic illnesses.

## 1 Introduction

As the world population ages, the prevalence of multimorbidity, defined as the coexistence of two or more chronic conditions, continues to rise (1, 2). In the United States, approximately 67% of Medicare beneficiaries live with multimorbidity, with prevalence increasing to over 80% among individuals aged 85 years and older (2, 3). Older adults with multimorbidity face substantial clinical challenges, including polypharmacy, conflicting treatment guidelines, fragmented care, and difficulties with treatment adherence (4–7). These complexities are associated with increased mortality, functional decline, reduced quality of life, and escalating healthcare utilization (8, 9). The heterogeneity inherent in multimorbidity emphasizes the need for a more individualized approach to care that considers the biological, physical, social, psychological, and spiritual dimensions of wellbeing among older adults.

Precision medicine has emerged as a transformative healthcare paradigm focused on tailoring prevention and treatment according to individual variability in genetics, biology, environment, and lifestyle (10, 11). Traditionally, precision medicine has emphasized molecular profiling, biomarkers, and genomics to optimize disease-specific interventions. While this approach has advanced care in areas such as oncology and pharmacogenomics, its application in older adults with multimorbidity remains challenging (11). For older adults with multimorbidity, particularly those living with serious illnesses, a solely biological focus overlooks the lived complexity of illness and the multidimensional nature of living with the burden of an illness. Suffering associated with multimorbidity is rarely limited to physical symptoms. Functional decline, uncertainty, loss of independence, existential distress, caregiver burden, and social isolation frequently accompany chronic disease (12).

Although precision medicine has gradually evolved toward a more holistic and patient-centered approach through the broader concept of precision care, incorporating physical, psychological, and social dimensions of health, spiritual needs remain largely underrecognized and insufficiently integrated within dominant precision medicine discourse, representing a critical gap in achieving truly individualized care (13). Evidence shows that spiritual well-being correlates with improved mental health (less anxiety, depression), reduced symptom burden, higher life satisfaction, and better end-of-life outcomes including potential for longevity (14, 15).

For many older adults, spirituality becomes central to how they experience illness and make meaning of suffering, particularly within palliative care contexts (15). Importantly, palliative care is not limited to end-of-life situations but is also relevant in the management of multimorbidity, at any stage of serious illness and may be delivered alongside curative therapies (16). In these contexts, clinical decision-making often involves balancing trade-offs among longevity, functional status, symptom burden, and quality of life (17). This paper argues that integrating precision medicine into palliative care requires a whole-person approach that recognizes and addresses the often-neglected spiritual needs of older adults with multimorbidity. By attending not only to the biological aspects of illness but also to patients’ emotional, existential, and spiritual needs, healthcare providers can deliver truly patient-centered and individualized whole-person care.

This article examines the relevance of precision medicine in the care of older adults with multimorbidity from a palliative and whole person approach, reviews the evidence linking spirituality to health outcomes, proposes an integrated framework for whole-person precision care, and discusses clinical, ethical, and implementation considerations.

## 2 Integrating precision medicine into palliative care of older adults with multimorbidity

Precision medicine is an innovative approach that uses an individual's genomic, environmental, and lifestyle information to personalize preventive, diagnostic, and therapeutic strategies (18). Precision medicine has several applications in palliative care, including pharmacogenomic testing to personalize pain and symptom management, real-time patient monitoring, and AI-driven tools that predict disease progression and trigger early palliative care referrals (19–21). Such proactive approaches are particularly important for older adults with multimorbidity, who often have more complex treatment needs and are at higher risk of complications (22, 23).

Therefore, in the care of older adults with multimorbidity, precision medicine should be delivered in such a way that the patient's goals, function, and quality of life guides treatment decisions safely and effectively (24). While the current strategies of utilizing genomic data and predictive algorithms may improve clinical precision, they fall short of addressing all the dimensions of suffering associated with multimorbidity particularly when a serious illness is involved (25). For this reason, integrating precision medicine into palliative care requires a whole-person framework grounded in the biopsychosocial-spiritual model (26). Within such a framework, the often-overlooked spiritual care becomes an essential component rather than an afterthought.

We propose that achieving meaningful precision care of complex patients requires consideration of measurable psychosocial and spiritual characteristics that influence health behaviors, treatment preferences, and outcomes. A precision whole-person care approach is not intended to replace existing frameworks, such as patient-centered care or the biopsychosocial-spiritual model, but rather to complement and strengthen them. Whereas these frameworks identify the key dimensions of the person that should be considered in care, a precision approach emphasizes their systematic assessment and translation into actionable, individualized care plans. Importantly, a precision approach does not presume that spiritual care is relevant for every patient; instead, it helps identify those for whom spiritual concerns are clinically meaningful and connect identified needs with appropriate resources.

## 3 Spirituality as a domain of precision

Spirituality, broadly defined as the search for meaning, purpose, and connection beyond self, is deeply important for many older adults (27). It is an important component of the biopsychosocial-spiritual framework (26). Spirituality represents a fundamental dimension of human variability that influences health behaviors including treatment preferences, coping, resilience, as well as biological outcomes such as stress response and immune function (15, 28). Recognizing the spiritual dimension of humanity allows clinicians to better understand patients' values and beliefs influencing their health choices; support mental wellbeing by reducing stress and anxiety; and foster resilience and hope, which can improve other clinical outcomes including end of life outcomes (15, 29, 30). In one cross-sectional Portuguese sample, spirituality was the only variable to have a direct effect on mental quality of life in older adults with chronic diseases (31). However, the study's cross-sectional design and single-country sample limit causal inference and broader cross-cultural generalizability. Although causality remains complex and study designs are heterogeneous, the cumulative evidence supports the notion that spirituality is a relevant dimension of holistic care in older adults (32). Routine spiritual assessment using tools (e.g., FICA, HOPE, or Meaning-Centered approaches) allow clinicians to identify existential suffering and tailor interventions (33). Incorporating structured spiritual assessment enhances identification of existential distress and enables targeted interventions such as chaplaincy and meaning-centered therapies (34). This approach may be conceptualized as “values phenotyping,” the systematic identification of what matters most to patients and the integration of these values into clinical decision-making. This aligns with the concept of phenotyping in precision medicine, which involves a detailed and comprehensive analysis of a patient's clinical, molecular, and imaging characteristics to guide tailored treatment (35). By integrating the spiritual dimension into patient care, precision medicine can move beyond being solely biologically precise toward becoming more value-concordant, with treatment decisions guided by individuals' values, beliefs, and sources of meaning. Chaplaincy-supported models can help translate patients' spiritual priorities into actionable care goals, complementing genomic and clinical data with a deeper understanding of the person behind the patient (36). In this way, spirituality helps operationalize the “whole-person” ideal of precision medicine, ensuring that care for older adults with multimorbidity addresses biological, psychosocial, and existential complexity with equal rigor.

## 4 Framework for whole person precision care

The framework proposed here integrates the principles of precision medicine within the biopsychosocial-spiritual model of health to support whole person care in older adults with multimorbidity. Multimorbidity reflects complex interactions among biological vulnerability, psychological resilience, social context, and spirituality (37). This framework operationalizes whole-person care through a multidimensional assessment and intervention strategy that incorporates all the domains of health into clinical decision-making (Table 1). By systematically integrating these domains into precision care pathways, clinicians could develop individualized care plans that align medical treatment with patients' functional status, personal values, and life contexts.

**Table 1:** Whole-person precision care framework.

Domain	Key elements	Validated Instrument/Measure	Measurable/Actionable Component of Domain	Clinical Application
Biological	Genomics, Biomarkers, Phenotyping	Disease-specific biomarkers/genomic measures	Biomarkers and Phenotypes identify biologic risk and potential treatment targets	Risk stratification, treatment selection, precision therapeutics
Functional	Frailty, ADLs/IADLs, mobility	Clinical Frailty Scale (38), Katz ADL (39), Lawton IADL (40)	Extent of Frailty, Mobility, and Dependence identifies modifiable functional limitations	Rehabilitation, care planning, fall prevention, preserve independence
Psychosocial	Mental health, coping, social support, loneliness	PHQ-9 (41) GAD-7 (42), UCLA Loneliness Scale (43)	Standardized measure of depression, anxiety, and social isolation that can trigger behavioral and social interventions	Behavioral health referral, social prescribing, caregiver/social support interventions
Spiritual	Meaning, purpose, beliefs, spiritual well being/distress	HOPE (44), FICA (45)	Identification of spiritual well-being, meaning/peace, faith, unmet spiritual needs to guide individualized spiritual care	Chaplain referral, meaning centered interventions, team based spiritual care
Goals of Care	Values, preferences, priorities, trade-offs	Patient Priorities Care (PPC) (46), Goals of Care forms, structured values assessment	Elicits what matters most, healthcare preferences, treatment burden patient is willing to accept	Priorities aligned treatment decisions, goal concordant shared decision-making
Prognosis	Disease trajectory, life expectancy, risk of decline	Schonberg Index for community dwelling older adults (47)	Generates estimates of mortality risk helpful for contextualizing expected benefits and burden of preventive or disease directed treatment approaches	Advanced care planning, treatment evaluation, end-of-life discussions and medical decisions

### 4.1 Multidimensional assessment

A multidimensional approach to clinical assessment provides a more complete understanding of the individual patient, helping clinicians develop personalized, goal-concordant, and holistic care plans that address the complex and interconnected needs of older adults living with multimorbidity. The multidimensional assessment includes biological and phenotypic profiling, psychosocial and spiritual profiling, assessment of patient values and goals, and evaluation of contextual, environmental, and lifestyle factors. Given the extensive literature on biological and phenotypic approaches to precision medicine, its respective subsection provides only a focused overview, while greater emphasis is placed on the less well-characterized psychosocial and spiritual dimensions that are central to the proposed expanded framework.

#### 4.1.1 Biological and phenotypic profiling

Building on established precision medicine principles, biological and phenotypic characterization involves patient characterization by assessing genomics, biomarkers, comorbidity networks, frailty indices, and functional measures such as gait speed and activities of daily living (ADL) (48, 49). These measures capture biological heterogeneity and functional vulnerability that are particularly relevant in older adults with multimorbidity. One of the most established applications is pharmacogenomics and precision prescribing. Older adults with multimorbidity are particularly vulnerable to adverse drug reactions, and medication interactions. Genomic testing is increasingly used to identify genetic variations that influence drug metabolism, allowing clinicians to tailor medication selection and dosing to individual patients. For example, variants in cytochrome P450 enzymes may influence how patients metabolize opioids, antidepressants, or antipsychotics commonly used in palliative symptom management (50).

#### 4.1.2 Psychosocial and spiritual profiling

Biological and phenotypic data should be complemented by structured assessment of psychological, social, and spiritual factors, including screening for existential distress, using validated instruments. In serious illness, assessment should explicitly include prognosis, symptom burden, functional trajectory, and acceptable trade-offs. Psychological well-being, coping capacity, social relationships, and sources of meaning significantly influence how older adults experience illness and respond to treatment (15, 51). Healthcare providers should therefore routinely screen for psychosocial stressors and spiritual needs as part of holistic patient evaluation (32). This allows clinicians to identify patients experiencing existential distress or spiritual suffering early in the course of illness management. It also ensures that spirituality is assessed with the same clinical rigor as other patient characteristics. Simple and validated tools such as FICA (Faith, Importance, Community, Address in care), HOPE (Hope, Organized religion, Personal spirituality, Effects on care), and NIH-HEALS can be integrated into standard clinical workflows with healthcare providers trained to efficiently use these tools to reduce significant time burden in conducting spiritual assessment. These tools help to promote open dialogue about meaning, coping, and sources of strength in a manner that respects each patient's background and beliefs (33). Importantly, some of these screenings such as spiritual screening may be conducted by nursing staff or medical assistants during intake, with follow-up by clinicians or chaplains as appropriate. Documenting psychosocial as well as spiritual needs of older adults with multimorbidity could provide an entry point for more holistic care planning and timely chaplaincy referral.

#### 4.1.3 Assessment of patient values, goals, and priorities

Values and goals operate at different but complementary levels. Values reflect enduring beliefs about what makes life meaningful, such as self-sufficiency, dignity, connectedness, or quality of life, whereas goals are more specific and context-dependent outcomes, such as attending a family event or remaining at home for as long as possible. Values are the reasons behind the goals people set. For many older adults, priorities may include maintaining independence, preserving cognitive and physical function, minimizing symptom burden, remaining socially connected, achieving spiritual fulfillment, or avoiding repeated hospitalization and treatment burden (52). Assessing patient values and goals is a critical component of multidimensional assessment in older adults with multimorbidity, as it helps determine which clinical findings and precision-based interventions are most meaningful to the individual patient. This approach shifts care from a disease-centered model toward goal-concordant care, ensuring that treatment decisions align with what matters most to the patient rather than focusing solely on disease-specific outcomes. Within precision and palliative care models, identification and clear documentation of patient's values and priorities serve as a guide for shared decision-making, treatment prioritization, and even deprescribing when interventions no longer align with patient-defined goals or quality-of-life priorities. Tools such as the *What Matters Most–Structured Tool* (WMM-ST), *Patient Priorities Care* (PPC), and *Goal Attainment Scaling* (GAS) help clinicians translate patient preferences into realistic and actionable care goals (53). Within precision and palliative care models, these values could serve as a guide for shared decision-making, treatment prioritization, and deprescribing when interventions no longer align with patient-defined goals or quality-of-life priorities. Ultimately, centering care around patient values could promote more compassionate, individualized, and holistic care for older adults living with multimorbidity.

#### 4.1.4 Assessing contextual and lifestyle factors

Contextual assessment includes evaluating socioeconomic status, living environment, health literacy, social support systems including caregiver availability. These factors significantly influence disease progression, treatment adherence, functional status, and quality of life. all of which shape a patient's ability to navigate complex healthcare systems and manage chronic illness (54). Environmental factors like housing conditions, neighborhood safety, accessibility, mobility, and exposure to pollution or green spaces affect physical and psychological well-being in older adults. Supportive environments may promote independence, physical activity, and cognitive health, whereas unsafe or restrictive environments may contribute to functional decline and emotional distress (55). Lifestyle factors including diet, physical activity, smoking, alcohol use, sleep quality, stress, and medication adherence should likewise be assessed, as many are modifiable contributors to multimorbidity and symptom burden. Several multidimensional assessment frameworks may support this process, including the Comprehensive Geriatric Assessment (CGA), the Geriatrics 5Ms Framework, and the Older Americans Resources and Services (OARS) assessment tool. These models facilitate a more holistic understanding of patient needs by integrating medical, functional, psychological, social, and personal domains into care planning (56).

### 4.2 Personalized care plans

Findings from multidimensional assessment guide the development of an individualized care plan that brings together biological, psychosocial, spiritual, and contextual considerations in clinical decision-making. Management is shaped not only by disease-specific factors and biomarkers but also by prognosis, symptom burden, functional status, frailty, and patient-defined goals of care, with careful attention to avoiding harm and maintaining quality of life. Psychological, social, and spiritual concerns identified during the assessment are addressed through appropriate support, including behavioral health services, social care resources, and chaplaincy involvement where needed (57). Care plans are grounded in the patient's expressed values and goals, so that clinical decisions reflect priorities such as independence, symptom relief, and overall well-being (58). This process relies on ongoing conversations with patients and, when appropriate, their caregivers to ensure that decisions remain aligned with what matters most to them. Care planning is not static. It requires regular review and adjustment as health status, circumstances, and preferences change over time. Care plans should prioritize symptom relief, quality of life, dignity, and avoidance of non-beneficial interventions. Advance care planning should be considered a core precision intervention. Approaching care in this manner supports trust, encourages engagement, and helps ensure that patients feel respected and understood (59).

### 4.3 Engaging multidisciplinary teams

Whole-person care requires collaboration across disciplines. Chaplains, social workers, psychologists, and primary care clinicians each contribute complementary expertise (36, 56). Primary care clinicians and specialists guide diagnosis and medical management, while nurses play a central role in care coordination, monitoring, and patient education. Pharmacists contribute to medication review and optimization, particularly in the context of polypharmacy. Social workers and psychologists address psychosocial needs, including mental health, coping, and access to community resources, while rehabilitation professionals support functional ability and independence. Chaplains as trained spiritual care specialists, play a distinctive role in facilitating meaning-centered conversations, supporting families during transitions, and helping clinicians navigate ethically or spiritually complex situations (56). Effective integration of these roles requires structured collaboration through interdisciplinary case discussions, shared care plans, and clear communication pathways. Such models help to reduce fragmentation of care, a common challenge in patients with multimorbidity and frailty (60). Although access to social workers and psychologists in outpatient settings is expanding, a critical gap remains in the availability of healthcare chaplaincy services, particularly in primary care clinics where longitudinal care plans are developed. Integrating chaplains into outpatient settings will require addressing key system-level barriers, including reimbursement models that do not routinely compensate spiritual care or time spent addressing goals and values, limited chaplain staffing relative to patient needs, and competing demands on clinicians’ time.

Evidence from interdisciplinary care programs in older adults with multimorbidity shows that coordinated, team-based approaches incorporating comprehensive assessment, medication review, and proactive management can significantly reduce hospitalizations and emergency visits (61). Findings from geriatric oncology and palliative care further support the value of multidisciplinary teams in managing complex patients These approaches improve quality of life, enhance symptom control, and allow care to be better aligned with patient goals by integrating functional, psychosocial, and supportive care domains into clinical decision-making (62). Multidisciplinary involvement also improves clinicians' ability to address non-physical dimensions of illness, including psychological and spiritual concerns, which are often under-recognized in routine care (63). Overall, a well-coordinated multidisciplinary model enables more comprehensive, consistent, and patient-centered care, ensuring that biological, functional, psychosocial, and spiritual needs are addressed in an integrated manner (Figure 1).

**Figure 1:**
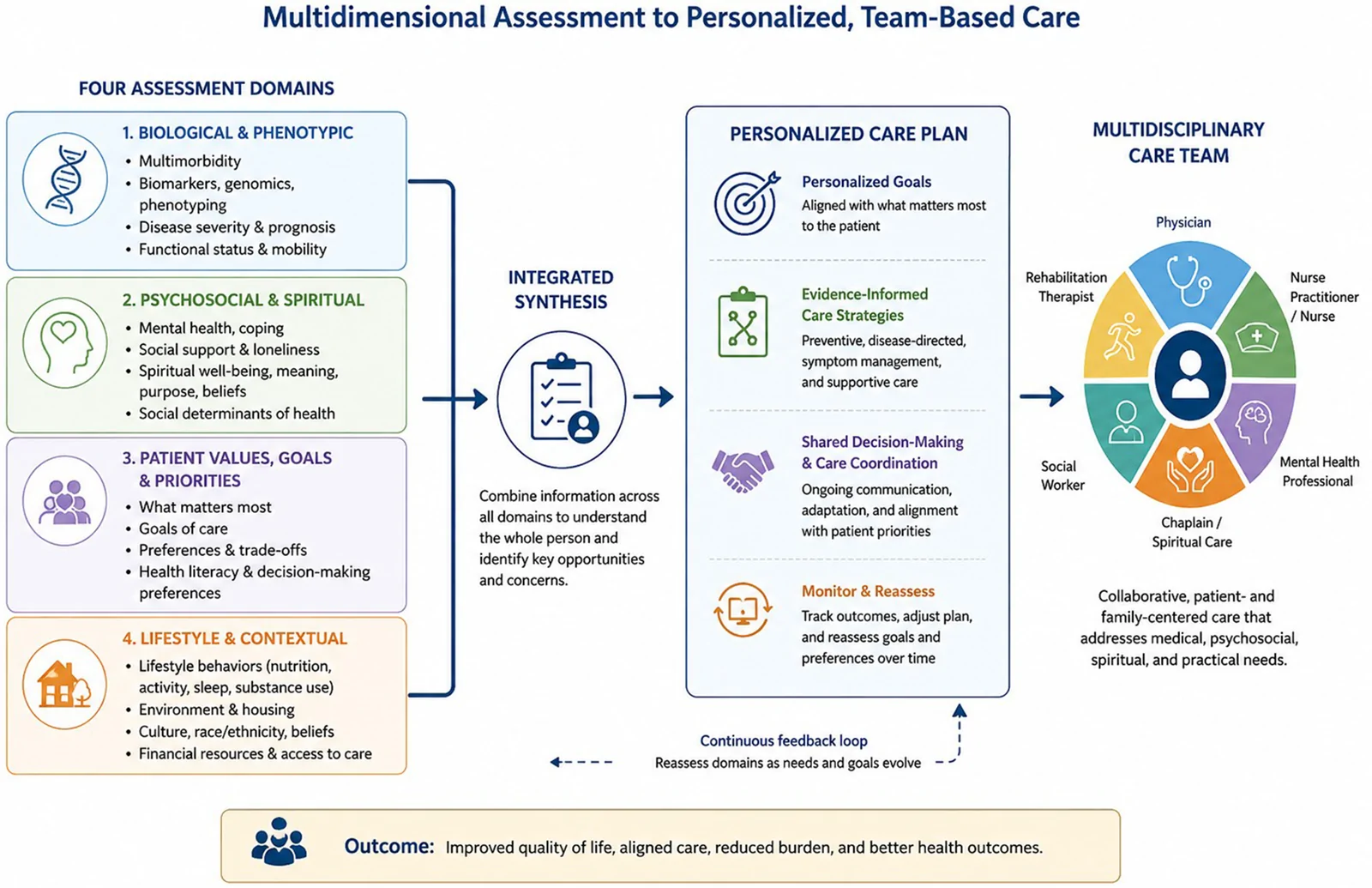
A multidimensional and multidisciplinary framework integrating precision and palliative care approaches for older adults with multimorbidity. Image source: Figure conceptualized by the authors and generated using ChatGPT (OpenAI), 2026.

### 4.4 Educating providers

Delivering whole-person care for older adults with multimorbidity requires clinicians to be trained beyond disease-specific management to include functional, psychosocial, cultural, and contextual dimensions of care (64). However, many clinicians report limited preparation in these areas, particularly in addressing complexity, eliciting patient goals, and engaging in person-centered communication (65). Clinicians also frequently report discomfort or lack of training in discussing spirituality with patients, often citing concerns about appropriateness, time, or perceived boundaries (66). Incorporating spiritual care training into medical and allied health education through workshops, communication simulations, and reflective exercises, could increase comfort and skill in addressing spirituality as a normal component of care (67).

Educational programs should emphasize cultural humility, shared decision-making, and effective teamwork across disciplines. Embedding these competencies into routine training supports a broader approach to care that reflects patients lived experiences and values (68).

### 4.5 Leveraging technology

Technology plays an increasingly important role in the integration of precision medicine and palliative care. Advances in genomics, artificial intelligence, digital health platforms, and electronic health records are transforming how supportive care is delivered to older adults with multimorbidity. These technologies allow clinicians to identify patient needs earlier, personalize interventions, and monitor symptoms more continuously and accurately than traditional models of care (19). However, digital health technologies should extend beyond biomarker monitoring to include patient-reported outcomes such as symptom burden, quality of life, and spiritual well-being, particularly for older adults with complex needs (69). Telehealth platforms may improve access to care for patients with mobility limitations or those living in underserved areas, while also enabling coordinated participation from multiple members of the care team (70). Tele-palliative care and tele-chaplaincy models enable delivery of precision care into the home. Beyond improving access, the expanding role of digital health offers new avenues to support spiritual well-being, particularly for homebound or socially isolated older adults. Emerging models such as tele-chaplaincy, virtual support groups, and patient portals facilitate access to spiritual care when in-person engagement is not feasible (71). Incorporating spiritual health assessments into electronic health records (EHRs) enables clinicians to document and track spiritual needs alongside physical and mental health indicators, contributing to a more comprehensive understanding of patient well-being (72). Additionally, mobile health applications designed to promote mindfulness, reflection, or connection with faith communities may be used as adjuncts to clinical care, extending the reach of spiritual support beyond traditional healthcare settings (73). Importantly, technology-delivered spiritual care must be implemented with attention to health equity. Older adults with limited digital literacy, poor access to internet, technological, or socioeconomic resources may be disproportionately excluded from telehealth and tele-chaplaincy approaches. Prior to implementing digital platforms for delivering spiritual care, healthcare providers should assess patients' digital access, capabilities, cultural context, preferences and offer flexible modalities, including in-person, telephone, and technology-enabled care. Such an approach is essential to ensure that digital innovation expands access to spiritual care rather than exacerbating existing disparities. Collectively, these innovations may promote continuity of care and reinforce the inclusion of spiritual well-being as a core component of health within a precision medicine framework.

## 5 Ethical considerations

Integrating spirituality into whole-person precision care requires heightened ethical vigilance to ensure that care remains patient-centered, respectful, and non-coercive. Of the ethical considerations, foremost is respect for patient autonomy, which requires that any assessment or intervention be guided by the patient's preferences, with explicit consent and the option to decline. Spiritual care as part of whole person care should function as a supportive resource rather than a directive component of treatment, aligning care with what matters most to the individual (74). Equally important is the need to uphold cultural and religious diversity. Patients represent a wide spectrum of spiritual, religious, and secular perspectives, and clinicians must approach each encounter with cultural humility, avoiding assumptions and allowing patients to define their own beliefs and values. Incorporating spirituality ethically therefore depends on individualized assessment rather than standardized or imposed frameworks (25). A critical ethical boundary is the avoidance of coercion. Clinicians and care teams must not introduce or promote personal beliefs but instead create space for patients to express their own spiritual concerns when relevant. When spiritual care is offered, it should be facilitative, supporting coping, meaning making, and decision-making, without creating perceived pressure (34). Ethical integration also requires sensitive spiritual assessment and appropriate use of information. Documentation of spiritual and religious information in the EHR raises important ethical considerations related to privacy, confidentiality, and potential for bias or discrimination. Therefore, information on spiritual history documented in EHR should be clinically relevant and voluntarily shared by patients. Spiritual histories should be obtained with transparency and consent, treated as confidential, and used solely to inform patient-centered care planning. Referral to trained spiritual care providers, such as chaplains, can further ensure that patients receive specialized support when desired, while maintaining professional boundaries within clinical practice (75). When approached appropriately, spirituality can enhance holistic care while preserving the integrity, inclusivity, and ethical standards of clinical practice (76). True precision requires proportionality, that is, aligning interventions with patient goals, expected benefit, and burden.

## 6 Future research directions

There is a critical need for more research designed toward empirical evaluation of whole-person precision care in older adults with multimorbidity. First, prospective studies are needed to validate real world implementation of the biopsychosocial-spiritual framework across primary care and palliative care settings, to determine its feasibility, acceptability, and clinical utility. Such studies should examine whether multidimensional assessment improves identification of unmet psychosocial, existential, and spiritual needs and facilitates more individualized, goal-concordant care. Second, longitudinal studies should determine whether systematic assessment and management of psychosocial and spiritual needs translate into clinically meaningful outcomes that have remained understudied, including functional status, treatment burden, and healthcare utilization. Lastly, implementation studies should evaluate models for integrating healthcare chaplains into longitudinal care of older adults with multimorbidity in ambulatory and homebased settings. More evidence is needed regarding optimal referral pathways, workforce requirements, cost effectiveness, and sustainability of chaplaincy integration, including whether earlier identification and management of spiritual distress influences potentially avoidable hospitalization, promotes satisfaction with care, or improves quality of life and survival.

## 7 Conclusion

The integration of precision medicine into palliative care represents an important paradigm shift toward more individualized and holistic care for older adults with multimorbidity. Through advances in genomics, predictive analytics, artificial intelligence, and digital health technologies, clinicians are increasingly able to tailor supportive care according to each patient's unique biological and clinical profile. However, the complexity of multimorbidity extends beyond biological dysfunction alone and requires approaches that address the psychological, social, environmental, and spiritual dimensions of illness. This is particularly vital for older adults with multimorbidity, who often face complex medical, emotional, and existential challenges. To achieve true precision in healthcare for older adults particularly, those with multimorbidity involving a serious illness, it must go beyond the biomedical and physical dimensions to encompass the more complex psychosocial and spiritual contexts that shape each person's total experience of illness, decision-making, and healing. Applying a biopsychosocial-spiritual framework to precision palliative care enables healthcare providers to move beyond disease-centered treatment toward truly person-centered care. Systematically screening for spiritual needs, aligning care with patients' beliefs, leveraging multidisciplinary expertise, educating providers, and utilizing technology together form a comprehensive strategy for addressing the spiritual dimension of health. Ultimately, the goal is not simply to prolong life, but to improve the lived experience of illness and ensure that older adults with multimorbidity receive care that reflects the full complexity of their humanity.

## Data Availability

The original contributions presented in the study are included in the article/Supplementary Material, further inquiries can be directed to the corresponding author/s.
